# Bartlett on Fevers

**Published:** 1852-09

**Authors:** 


It is for sale by Keen & Brothers, Chicago. J.

*Bartlett on Fevers.*

We have received from the publishers, Blanchard & Lea, Philadelphia, the third edition of Dr. Bartlett's very excellent work on the history, diagnosis and treatment of the fevers of the United States. This book, we presume, is a familiar acquaintance of our readers. In the preface the author says, "Dr. Jenner's researches have enabled me to add largely to the fullness and completeness of the description of typhus fever; and I have availed myself liberally of his facts and arguments in elucidation of the important question of the true relation to each other of the two great forms, or species, of continued fever." The chief value of the book does not consist in its mechanical execution, although this is in the publishers' best style. It will continue to be as it has been, a favorite with the profession. It is for sale by Keen & Brothers, Chicago.

